# *Rosa × alba*: source of essential minerals and volatile oils

**DOI:** 10.1007/s13659-011-0012-x

**Published:** 2011-09-16

**Authors:** Karim Hosni

**Affiliations:** Laboratoire des Substances Naturelles, Institut National de Recherche et d’Analyse Physico-chimique (INRAP), Pôle technologique de Sidi Thabet, 2020 Ariana, Tunisia

**Keywords:** *Rosa × alba*, Rosaceae, proximate composition, essential minerals, volatile oil constituents

## Abstract

*Rosa × alba* is cultivated almost exclusively for the production of aromatic water and fruits widely used as ingredients in some traditional food preparations sold commercially. Results of the proximate and elemental analyses revealed that flowers exhibited higher moisture content while the dry matter content was higher in leaves. Total fat and ash was higher in fruits when compared with leaves and flowers. All studied organs were found to be rich in essential mineral such as K, Ca, P and Mg. Qualitative and quantitative analysis of the volatile oils revealed different composition patterns between organs. Linalool and geraniol were found as major constituents of the leaf oil whereas 2-phenethyl alcohol and eugenol were the major constituents of the floral oil. In contrast, fruits showed very distinct composition and alkanes/alkenes were found to have the major contribution. The present composition could justify the traditional use of *Rosa × alba* which could be considered as a potential source of essential minerals and volatile constituents.

## Introduction

The Rosaceae family is one of the most employed as a consolidated source of phytoproducts with functional properties[1]. Within this family, the genus *Rosa* provides various species and their essential oils possess a wide range of applications as flavour, fragrance and additive in cosmetic and toiletries[2]. In addition to their aromatic composition, many *Rosa* species from all over the word have been evaluated for their food-related biological properties and a multiple functional uses have been suggested. For example, teas made from the fruits of *Rosa canina* have mild laxative and diuretic tendencies[1]. They have also been used for the prevention and the treatment of common cold, influenza-like infections, infectious diseases, for vitamin C deficiency, fever, general exhaustion, gastric spasms, prevention of gastritis and gastric ulcers, diarrhea, for gallstones and gallbladder discomforts, urinary tract diseases and discomforts, for inflammatory disorder, arthritis, nephritis, rheumatism, gout, sciatica, diabetes, inadequate peripheral circulation and lung ailments[3,4]. Mixed with small amount of vinegar, rose hips were used as antidote for the treatment of iron toxicity as reported by Saad et al.[5].

For nutritional purposes, fruits are used for the production of marmalade, jam, dessert soup, wine and juices[3,6]. Ground in a hand mill and cooked with milk, they could be used as children's snack and baby food as reported by the latter authors.

The functional properties of some *Rosa* species were attributable to a wide array of bioactive ingredients such as minerals, fatty acids, volatile oils, ascorbic acid, phenols, flavonoids, tannins and sugar[7–11]. Despite the substantial data on the phytochemistry of some *Rosa* species namely *R. canina,* there are a dearth of information about the chemical constituents of *Rosa* x *alba*.

In Tunisia, *Rosa x alba,* known as 'Nesri' is used for the production of aromatic water called 'Nesri water'. The latter, usually obtained by hydrodistillation of the flowers is highly appreciated and consumed as health-promoting product as well as to prevent cardiovascular diseases, although no pharmacological investigations have supported this application so far. Additionally, the 'Nesri water' is widely used as a flavouring agent of drinks, for the production of jam, marmalade and cake.

Despite the great socio-economic relevance of *Rosa x alba*, Tunisian native species have not received much interest. One of the major limitations to a better exploitation of this species is our still incomplete knowledge of its chemical composition. Therefore, any study concerned with the phytochemistry of this species is considered crucial to define the possible application areas for the rational use of this species.

With regard to this topic, this study aims to provide the proximate, mineral and volatile oil composition of different organs of *Rosa x alba* which has not been reported to date.

## Results and Discussion

**Proximate Composition and Mineral Content.** The proximate analysis and mineral contents of *Rosa x alba* leaves,

*To whom correspondence should be addressed. E-mail: karim.hosni@inrap.rnrt.tn

**Table 1. Proximate composition and mineral contents in leaves, flowers and fruits of *Rosa x alba*.**

| **chemicals (%)** | **leaves** | **flowers** | **fruits** |
|---|---|---|---|
| moisture | $50.9^{c*} \pm 4.45$ | $93.7^{a**} \pm 6.32$ | $66.4^{b} \pm 6.17$ |
| total dry matter | $49.1^{a} \pm 3.31$ | $6.3^{c} \pm 0.56$ | $33.6^{b} \pm 3.79$ |
| total fat | $0.6^{b} \pm 0.04$ | $1.1^{a} \pm 0.06$ | $1.6^{a} \pm 0.04$ |
| ash | $7.3^{b} \pm 0.61$ | $5.4^{bc} \pm 0.04$ | $9.7^{a} \pm 0.06$ |
| **minerals (ppm)** | | | |
| Na | $0.7^{c} \pm 0.03$ | $1.6^{a} \pm 0.09$ | $1.2^{b} \pm 0.13$ |
| P | $3829^{c} \pm 101.7$ | $4356^{b} \pm 243.17$ | $5129^{a} \pm 491.07$ |
| K | $4713^{b} \pm 183.87$ | $4921^{b} \pm 273.12$ | $5761^{a} \pm 156.23$ |
| Mg | $938^{b} \pm 49.81$ | $1084^{a} \pm 65.45$ | $1137^{a} \pm 76.49$ |
| Fe | $29^{a} \pm 2.31$ | $26.4^{a} \pm 1.27$ | $27.8^{a} \pm 2.43$ |
| Ca | $1920^{a} \pm 55.72$ | $1688^{b} \pm 103.43$ | $1743^{b} \pm 63.71$ |
| Cu | $9.6^{b} \pm 1.73$ | $13.4^{a} \pm 0.55$ | $12^{a} \pm 0.32$ |
| Mn | $16.3^{b} \pm 2.12$ | $19.7^{a} \pm 1.53$ | $18.2^{a} \pm 2.86$ |

Note: * Values within the same line followed by different superscripts are significantly different at $p < 0.05$; ** Values are means of triplicates ± standard deviation.

flowers and fruits are given in Table 1. As shown, the leaves were lower in moisture content (50.9%) when compared with flowers (93.7%) and fruits (66.4%). However, leaf total dry matter (49.1%) was significantly ($p < 0.05$) higher than those observed for flowers (6.3%) and fruits (33.6%). For total fat content, fruits showed the highest content (1.6%), about 300 and 150% higher than flowers and leaves, respectively. The same pattern was also observed for total ash content (9.7% in fruits versus 7.3 and 5.4 in leaves and flowers, respectively). The high content of ash was the result of high content of minerals mainly P, K, Ca and Mg. Other minor elements such as Fe, Cu and Mn were also found with appreciable amounts.

Overall, all parts of *Rosa x alba* could be used as unconventional sources of minerals since they contain an appreciable amount of essential macro and microelements such as Ca, P, Mg, K and Fe. These essential minerals have critical importance as a structural components in tissues and function in cellular and basal metabolism as well as water and acid-base balance (i.e. Ca is the main constituents of bone and teeth and has a pivotal role in cell membrane and muscles functioning as well as the regulation of blood pressure[15]; Fe, Mg and P act as a cofactors and they are essential for numerous metabolic pathways[16].

Since there are no reports regarding the chemical constituents of *Rosa x alba*, we cannot compare our results with earlier works. Nevertheless, few reports regarding the mineral composition of some *Rosa* species have been published[3,7,8]. At this point, Demir and Özcan[7] studied the chemical composition of *R. canina* fruits collected in two distinct locations from Turkey and found that the dry matter content varied from 20.5 to 23.47%, total fat (1.2–1.6%) and total ash ranged from 6.48 to 7.35%. Six years later, report from the same country revealed that the dry matter content in the fruits of six *Rosa* species ranged from 33.85 to 40.35%[3]. By using different extraction methods, Szentmihályi et al.[8] reported that the total fat in *R. canina* seeds from Hungary ranged from 3.25 to 6.68%.

Concerning the mineral composition, study from Turkey reported that the fruits of *R. canina* contains Na (3.97–4.67 mg/kg), K (850.5–1023.9 mg/kg), P (1850–2200 mg/kg), Fe (59.4–72.9 ppm), Zn (3.69–4.51 ppm), Mn (22.4–44.8 ppm), Mg (162.7–183.9 ppm) and Ca (133.3–146.7 ppm)[7]. Another report from the same country showed that the fruit of different *Rosa* species contains P (4860–5360 ppm), K (5467–7700 ppm), Ca (1220–2867 ppm), Mg (990–1254 ppm), Fe (18–72 ppm), Cu (9–27 ppm), Mn (6–56 ppm) and Zn (18–42ppm)[3]. The mineral content of rose hip has been also reported in Hungarian specimen[8]. These authors determined that the seeds contain Al (12 ppm), B (7.54 ppm), Ca (7948 ppm), Cr (0.211 ppm), Cu (5.69 ppm), Fe (20.15 ppm), K (3786 ppm), Mg (1193 ppm), Mn (22.54 ppm), Na (12.28 ppm), P (1781 ppm), S (866.2 ppm) and Zn (10.84 ppm). In a recent paper, Özcan et al.[17] described the mineral composition of the decoction of rose hips and found that it contains Ag (0.03 ppm), Al (13.7 ppm), As (0.2 ppm), B (2.9 ppm), Ba (0.34 ppm), Ca (3479.3 ppm), Co (0.32 ppm), Mn (13.93 ppm), Na (280.1 ppm), Ni (0.67 ppm), P (856.2 ppm), Pb (0.17 ppm), Sr (4.05 ppm), Ti (5.56 ppm), V (0.15 ppm) and Zn (3.96 ppm).

**Volatile Oil Constituents.** The hydrodistillation of leaves, flowers and fruits yielded 0.03, 0.07 and 0.01% (w/w) respectively, of pale yellowish oil with pleasant smell. The very low yields seem to be typical to the genus *Rosa*[18].

The chemical composition of the essential oil is summarized in Table 2. As can be seen, 51 compounds accounting for 89.5, 96.3 and 71.2% of the total composition were identified in leaves, flowers and fruits, respectively. The alkanes/alkenes compounds made the highest contribution in the leaf and fruit oils (53.4 and 65.9%, respectively). Tetracosane, pentacosane, hexacosane and heptacosane were the major compounds in leaves while docosane and tetracosane were the dominant ones in fruits.

In flower, this fraction was reduced to 33.7% of the total oil with 1-heptadecene and nonadecane being the major components. The flower oil was characterized by its higher alcohols percentage (53.9%). Alcohols, reputed for their high odoriferous potential and, hence, the most valuable components for agro-food, cosmetic and fine perfumery industries, were mainly represented with 2-phenethyl alcohol and eugenol in flower and linalool in leaf oil. With respect to the oil from leaves and flowers, fruit oil contained smaller amounts of alcohols (1.5%).

In all investigated volatile oils, aldehydes, mono- and sesquiterpenes and other components were found in minor amounts. In general, different qualitative and quantitative composition as single components and obviously as group components for the leaf, flower and fruit oils suggests that they were structured by different metabolic pathways on one hand, and may have specific biological and/or ecological roles on the other hand. For example, the occurrence of *α* and *β*-unsaturated aldehydes, and in particular (*E*)-2-hexenal, (*E*)-2-heptenal and (*E, E*)-2,4-heptadienal recognized for their highly bactericidal and fungicidal activities could represent important defense phytochemicals against bacterial and fungic invasions[19]. The defensive role of nonanal and linalool has been also reported in *Citrus lemon*[20]. The biological role of some components identified in our oils such as 2-phenylethanol, eugenol and linalool was also reported[21] since they have been shown to be involved in the attraction of pollinators.

From practical standpoint, these components have found numerous applications in cosmetic and food industries since they were used to improve the shelf-life and safety of processed fruits[22].

**Table 2. Composition of the volatile oils (% peak area) of *Rosa x alba* leaves, flowers and fruits.**

| No | compound | RI* | RI** | leaves | flowers | fruits |
|---|---|---|---|---|---|---|
| 1 | (*E*)-2-hexenal | 853 | 1231 | $1.2^{a***} \pm 0.03$ | | |
| 2 | (Z)-3-hexenol | 858 | 1391 | | $0.1^{a****} \pm 0.01$ | |
| 3 | *α*-pinene | 940 | 1032 | $0.3^{b} \pm 0.01$ | $3.2^{a} \pm 0.17$ | $0.2^{b} \pm 0.01$ |
| 4 | (*E*)-2-heptenal | 943 | 1335 | $0.3^{a} \pm 0.01$ | | |
| 5 | 1-octen-3-ol | 978 | 1453 | | | $0.9^{a} \pm 0.06$ |
| 6 | *β*-pinene | 983 | 1118 | $0.1^{c} \pm 0.00$ | $0.6^{a} \pm 0.02$ | $0.3^{b} \pm 0.02$ |
| 7 | (*E.E*)-2.4-heptadienal | 1014 | 1508 | $0.1^{a} \pm 0.01$ | | |
| 8 | 1,8-cineol | 1033 | 1213 | $0.2^{a} \pm 0.01$ | $0.1^{b} \pm 0.1$ | $0.1^{b} \pm 0.00$ |
| 9 | linalool | 1088 | 1553 | $21.1^{a} \pm 1.79$ | $0.5^{b} \pm 0.03$ | $0.1^{c} \pm 0.00$ |
| 10 | nonanal | 1108 | 1400 | $2.0^{a} \pm 0.12$ | $0.3^{b} \pm 0.02$ | $0.2^{b} \pm 0.01$ |
| 11 | 2-phenethyl alcohol | 1117 | 1933 | $0.2^{b} \pm 0.00$ | $12.9^{a} \pm 1.43$ | $0.1^{b} \pm 0.00$ |
| 12 | nonanol | 1149 | 1668 | | $0.1^{a} \pm 0.00$ | $0.1^{a} \pm 0.00$ |
| 13 | terpinen-4-ol | 1178 | 1611 | | $0.1^{a} \pm 0.00$ | |
| 14 | fenchyl alcohol | | 1591 | | $0.2^{a} \pm 0.00$ | |
| 15 | myrtenol | 1196 | 1804 | | $0.3^{a} \pm 0.00$ | |
| 16 | *β*-cyclocitral | 1233 | | $0.1^{a} \pm 0.00$ | | |
| 17 | geraniol | 1255 | 1857 | $5.7^{a} \pm 0.11$ | $0.2^{b} \pm 0.01$ | |
| 18 | tridecane | 1297 | 1300 | $0.2^{a} \pm 0.02$ | | $0.1^{a} \pm 0.00$ |
| 19 | eugenol | 1356 | 2192 | $1.5^{b} \pm 0.06$ | $39.4^{a} \pm 2.47$ | $0.2^{c} \pm 0.00$ |
| 20 | tetradecane | 1394 | 1400 | $4.3^{a} \pm 0.12$ | | |
| 21 | *β*-caryophyllene | 1418 | 1612 | $0.5^{c} \pm 0.02$ | $2.3^{a} \pm 0.06$ | $1.1^{b} \pm 0.11$ |
| 22 | 7,8-dihydro-*β*-ionone | 1422 | | | $0.1^{a} \pm 0.00$ | |
| 23 | *α*-guaiene | 1440 | | | $0.4^{a} \pm 0.0$ | |
| 24 | *α*-humulene | 1452 | 1687 | | $0.2^{a} \pm 0.01$ | |
| 25 | allo-aromedendrene | 1458 | 1661 | $0.1^{a} \pm 0.00$ | $0.2^{a} \pm 0.01$ | |
| 26 | germacrene-D | 1480 | 1726 | $0.1^{b} \pm 0.00$ | | $1^{a} \pm 0.06$ |
| 27 | *β*-selinene | 1481 | 1742 | | $0.1^{a} \pm 0.01$ | |
| 28 | *β*-ionone | 1482 | 1958 | $0.8^{a} \pm 0.04$ | $0.3^{b} \pm 0.02$ | |
| 29 | bicyclogermacrene | 1495 | 1755 | $0.2^{a} \pm 0.01$ | | |
| 30 | *δ*-guaiene | 1499 | - | | $0.3^{a} \pm 0.02$ | |
| 31 | *γ*-cadinene | 1511 | 1773 | | $0.1^{a} \pm 0.00$ | |
| 32 | *δ*-cadinene | 1526 | 1776 | $0.5^{a} \pm 0.04$ | | |
| 33 | lauric acid | 1566 | 2503 | $1.1^{a} \pm 0.06$ | | $0.8^{a} \pm 0.03$ |
| 34 | caryophyllene oxide | 1569 | 2008 | | $0.5^{a} \pm 0.02$ | |
| 35 | hexadecane | 1600 | 1600 | | $0.1^{b} \pm 0.01$ | $2.4^{a} \pm 0.16$ |
| 36 | tetradecanal | 1611 | 1933 | | $0.2^{a} \pm 0.01$ | $0.2^{a} \pm 0.00$ |
| 37 | 1-heptadecene | 1679 | | $2.6^{b} \pm 0.12$ | $6.5^{a} \pm 0.12$ | $1.2^{c} \pm 0.16$ |
| 38 | heptadecane | 1700 | 1700 | | $0.5^{a} \pm 0.11$ | $0.4^{a} \pm 0.02$ |
| 39 | benzyl benzoate | 1759 | 2655 | $0.2^{a} \pm 0.01$ | | |
| 40 | octadecane | 1800 | 1800 | $1.6^{a} \pm 0.05$ | $0.1^{c} \pm 0.00$ | $0.3^{b} \pm 0.01$ |
| 41 | 3-eicosene | 1840 | | | $0.2^{a} \pm 0.02$ | $0.1^{b} \pm 0.00$ |
| 42 | 1-nonadecene | 1875 | | | $0.5^{a} \pm 0.02$ | |
| 43 | nonadecane | 1900 | 1900 | $4.7^{b} \pm 0.38$ | $6^{a} \pm 0.72$ | $2.4^{c} \pm 0.15$ |
| 44 | *n*-eicosane | 2000 | 2000 | $0.9^{a} \pm 0.02$ | $0.6^{b} \pm 0.04$ | $0.2^{c} \pm 0.00$ |
| 45 | *n*-heneicosane | 2100 | 2098 | $4.9^{a} \pm 0.08$ | $4.2^{b} \pm 0.12$ | $3.4^{c} \pm 0.04$ |
| 46 | docosane | 2203 | 2200 | $3.1^{b} \pm 0.28$ | $2.3^{c} \pm 0.09$ | $27.8^{a} \pm 2.1$ |
| 47 | tricosane | 2300 | 2300 | | $2.5^{a} \pm 0.06$ | $1.6^{b} \pm 0.04$ |
| 48 | tetracosane | 2398 | 2400 | $7.7^{b} \pm 0.09$ | $1.9^{c} \pm 0.04$ | $19.7^{a} \pm 0.89$ |
| 49 | pentacosane | 2497 | 2500 | $9.1^{a} \pm 0.72$ | $4.2^{b} \pm 0.15$ | $2.6^{c} \pm 0.05$ |
| 50 | hexacosane | 2600 | 2600 | $7.4^{a} \pm 0.21$ | $1.8^{b} \pm 0.06$ | $2.3^{b} \pm 0.05$ |
| 51 | heptacosane | 2700 | 2600 | $7.0^{a} \pm 0.12$ | $2.3^{b} \pm 0.02$ | $1.4^{c} \pm 0.01$ |
| | ***group components*** | | | | | |
| | alkanes/alkenes | | | $53.4^{b}$ | $33.7^{c}$ | $65.9^{a}$ |
| | alkohols | | | $28.7^{b}$ | $53.9^{a}$ | $1.5^{c}$ |
| | aldehydes | | | $3.5^{a}$ | $0.5^{b}$ | $0.4^{b}$ |
| | monoterpene hydrocarbons | | | $0.4^{b}$ | $3.8^{a}$ | $0.5^{b}$ |
| | sesquiterpene hydrocarbons | | | $1.4^{c}$ | $4.1^{a}$ | $2.1^{b}$ |
| | others | | | $2.2^{a}$ | $0.4^{c}$ | $0.8^{b}$ |
| | total | | | 89.5 | 96.4 | 71.2 |

Note: RI. Retention Index as determined on a (*) HP-5MS and (**) HP-Innowax columns using the homologous series of alkanes. ***Values within the same line followed by different superscripts are significantly different at $p < 0.05$. **** Values are means of triplicates ± standard deviation.

In summary, results of this study indicate that leaves, flowers and fruits of *Rosa x alba* are rich sources of essential minerals mainly Ca, K, P, Mg, Fe, Cu, Mn and Na. So, the leaves and flowers could be consumed as salads and/or decoctions, while fruits could be consumed freshly as a dessert or processed to jam. Qualitative and quantitative analysis of the volatile oils from different organs revealed different compositional patterns between leaves, flowers and fruits. Because of the abundance of some components with documented biological properties such as α and *β*-unsaturated aldehydes and alcohols such as eugenol, linalool in leaves and 2-phenylethanol in flowers, the leaf volatile oil could find applications in food industry whereas, those from flowers seemed more appropriates in cosmetic and perfume industries. From fundamental standpoint, this study has extended our knowledge on the proximate, mineral and volatile oil constituents of *Rosa x alba* which has not been yet assessed.

## Experimental Section

**Plant Material.** Leaves, flowers and fruits samples were handpicked from cultivated plants of *Rosa x alba* growing continuously in the dog rose biodiversity garden (Mograne, Tunisia; latitude 36° 26' (N); longitude 10° 05' (E); altitude 156 m above sea). Means annual precipitation and temperature are 502 mm and 17.9°C, respectively. The plants were cultivated without any chemical fertilizer. The harvested material was air-dried at room temperature (20 ± 2°C) and subsequently essayed for proximate, mineral and volatile oil composition.

**Proximate Composition and Mineral Content.** Moisture content, total dry weight, total ash, total fat and mineral composition were determined according to the AOAC[12] methods. The mineral composition was determined after digestion with $HNO_3$ and $H_2O_2$ at 200°C. Potassium and sodium contents were measured on a Gallenkamp model digital flame analyser (Loughborough, England). Calcium, magnesium, copper, manganese and iron were determined on a Philips PU 9100 model atomic absorption spectrometry (Cambridge, England). The absorbance at 680 nm of the yellow colour, obtained from the Barton reaction, using a Jenway 6300 type spectrophotometer (Essex, UK) was used for the determination of phosphorus content.

**Volatile Oils Extraction.** The volatile oils were isolated from raw materials (100 g) by conventional hydrodistillation for 3h. The hydrodistillation was performed by a simple laboratory Quikfit apparatus which consisted of a 2000 ml steam generator flask, a distillation flask, a condenser and a receiving vessel. The obtained distillate was extracted twice with *n*-pentane and dried over anhydrous $Na_2SO_4$[13]. The organic layer was then concentrated, at 35°C using a Vigreux column at atmospheric pressure and subsequently analyzed.

**Gas Chromatography Analysis.** Gas chromatography analyses were carried out on a Shimadzu HRGC-2010 gas chromatograph (Shimadzu Corporation, Kyoto, Japan) equipped with flame ionisation detector (FID), auto-injector AOC-20i and auto-sampler AOC-20s. An apolar column HP-5 (30 m × 0.25 mm, 0.32 $\mu$m film thickness) was used. The oven temperature was held at 50°C for 10 min then programmed at 2°C/min to 190°C. The injector and detector temperature were held at 230°C. The flow of the carrier gas (Nitrogen) was 1.6 mL/min, the split ration was 1:20 and the injection volume for all samples was 1$\mu$L.

**Gas Chromatography-Mass Spectrometry (GC-MS) Analysis.** Gas chromatography-mass spectrometry (GC-MS) analyses were performed on a gas chromatograph HP 6890 (II) interfaced with a HP 5973 mass spectrometer (Agilent Technologies, Palo Alto, Ca, USA) with electron impact ionization (70 eV). A HP-5MS capillary column (60 m × 0.25 mm, 0.25 $\mu$m film thickness) was used. The column temperature was programmed to rise from 40°C to 280°C at a rate of 5°C/min. The carrier gas was helium with a flow rate of 1.2 mL/min. Scan time and mass range were 1s and 50–550 m/z, respectively.

**Components Identification.** The volatile compounds were identified by comparison of their retention indices relative to ($C_7$–$C_{20}$) *n*-alkanes with those of literature and/or with those of authentic compounds available in our laboratory, and by matching their mass spectral fragmentation patterns with corresponding data (Wiley 275 Llibrary) and other published mass spectra[14] as well as by comparison of their retention indices with data from the Mass Spectral Library "Terpenoids and Related Constituents of Essential oils" (Dr. Detlev Hochmuth, Scientific consulting, Hamburg, Germany) using the MassFinder 3 software (www.massfinder.com). The relative percentages of the essential oil components were obtained by FID peak-area normalization, all relative response factors being taken as 1.

**Data Analysis.** The experiment and analytical determinations were carried out in triplicate. To verify the statistical significance of all parameters, the values of means ± S.D. were calculated. The one-way analysis of variance (ANOVA) using Duncan's multiple range test at the significance level of $p < 0.05$ was used to compare means.

**Open Access** This article is distributed under the terms of the Creative Commons Attribution License which permits any use, distribution, and reproduction in any medium, provided the original author(s) and source are credited.
